# Comparisons of social and demographic determinants of tobacco use in the Democratic Republic of the Congo

**DOI:** 10.1186/s12992-020-00593-0

**Published:** 2020-07-20

**Authors:** Brian Colwell, Kizito B. A. Mosema, Matthew S. Bramble, Jay Maddock

**Affiliations:** 1 Texas A&M School of Public Health, 1266 TAMU, College Station, TX 77843-1266 USA; 2 Biamba Marie Motombo Hospital, Masina I, Blvd. Lumumba, Kinshasa, Democratic Republic of Congo; 3 Center for Genetic Medicine Research, Children’s Research Institute, Children’s National Medical Center, 111 Michigan Avenue NW, Washington, D.C, 20010 USA

**Keywords:** Tobacco, Smoking, Snuff, Democratic Republic of the Congo, Demographic and health survey

## Abstract

**Background:**

Worldwide, tobacco use has caused over 100 million deaths in the twentieth century and is projected to cause death in up to one billion people in the twenty-first century. It is a leading cause of early death and disability in over 100 countries and accounts for over 11% of global deaths, disproportionately affecting low- and middle-income countries. The purpose of the study was to examine a variety of social determinants of tobacco use in the Democratic Republic of the Congo, including region, sex, ethnicity, education, literacy, wealth index and place of residence, to gain insights with regard to tobacco use among sub-national groups.

**Methods:**

This project was a secondary data analysis of the 2013–2014 Demographics and Health Survey (DHS) for the Democratic Republic of the Congo. Logistic regressions predicting smoking, use of snuff and smoking cigars or natural tobacco as dichotomous variables were conducted. Independent variables included age, educational level, religion, rurality, literacy, wealth index, occupation and ethnicity.

**Results:**

Tobacco use is highest among those with less education and low literacy. It was also highest among the working poor. Older age and living in larger cities were predictive of smoking, although the relationship between age and smoking was not linear. There was a strong linear effect for wealth. Being in a professional, technical or managerial position was highly protective against smoking while being engaged in services, skilled and unskilled manual labor, and the army had significantly greater odds of smoking.

**Conclusions:**

Data indicate that tobacco use in the DRC, as is common in the developing world, is heavily concentrated in the working poor with lower educational status. Higher educational status is consistently predictive of avoiding tobacco use. Additionally, examining only national-level data to ascertain tobacco use levels and patterns may lead to mistaken conclusions that can lead to inefficient and ineffective allocation of resources aimed at controlling tobacco use.

## Introduction/background

Worldwide, tobacco use has caused over 100 million deaths in the twentieth century and is projected to cause death in up to one billion people in the twenty-first century [7, 6]. Tobacco use remains a leading cause of early death and disability in over 100 countries around the world today, accounting for over 11% of global deaths in 2015 [8]. Tobacco-related health burdens, however, disproportionately affect developing countries, with up to 80% of global tobacco-related mortality today occurring those in low- and middle-income countries (LMICs) [7, 23, 6]. While tobacco control has largely been successful in the most highly-developed countries, tobacco companies have increasingly been directing marketing efforts towards developing countries [4, 15]. Within sub-Saharan Africa (SSA) alone, there has been a 70% increase in mortality in tobacco-related deaths from 1990 to 2016 [10]. As communicable diseases are increasingly brought under control in developing countries, the burden of non-communicable, chronic diseases is expected to rise.

As LMICs battle disease and death due to infectious organisms and environmental conditions, heavy marketing of tobacco has led to what has been described as a “protracted double burden of infectious and chronic disease,” with nations caught between what has been identified as the third (age of degenerative and man-made diseases) and fourth (age of delayed degenerative diseases) phase of an epidemiological transition [2]. These nations, though, have the unique opportunity to shorten the time necessary to bring about dramatic reductions in the portion of disease attributed to tobacco by implementing many of the lessons learned in other more developed countries. For example, many of these countries are already signatories to the Framework Convention on Tobacco Control [22]. This document, signed by 168 countries with 181 total parties, provides coordinated strategies to address tobacco production, distribution, sales and consumption, including demand reduction, supply reduction, environmental protection and research/surveillance activities [24]. Yet surveillance and intervention activities are expensive and often underfunded. The World Health Organization and the U.S. Centers for Disease Control and Prevention recommend that at least 10 % of total financial resources for health promotion initiatives be devoted to surveillance and evaluation, including tobacco control [21].

“There is an inextricable and pernicious relationship between tobacco and poverty. In many ways, tobacco and poverty are part of the same vicious cycle [14]..” The Democratic Republic of the Congo (DRC) represents an example of a lower-income country battling communicable disease and environmental contamination as well as struggling to address causes of chronic disease and death such as tobacco use. The DRC is estimated to be the fourth most populous country in Africa, with a population of over 80 million people. Among the least developed in the world, with some of the most remote communities in the world, data from the DRC can provide direction to tobacco control efforts in other underdeveloped countries.

The DRC also exemplifies the paucity of data for many LMICs and the difficulties of gathering and interpreting data that are available. We did not find other studies of social determinants of tobacco and/or other substance use in this country in recent literature.

The extant data and relevant studies have only been descriptive in nature and have not addressed any geographical differences or social determinants. While these are important and necessary to describe and frame the problem, additional analyses can help to examine other determinants of tobacco use that may be important leverage points in implementing interventions to address tobacco.

The purpose of this study was to examine a variety of social determinants of tobacco use in the Democratic Republic of the Congo, including region, sex, ethnicity, education, literacy, wealth index and place of residence. These determinants were also used to predict tobacco use among a variety of groups. Additionally, we also examined sub-national patterns of use, using provincial data from a large national dataset.

## Methods

### Data

**Fig. 1:**
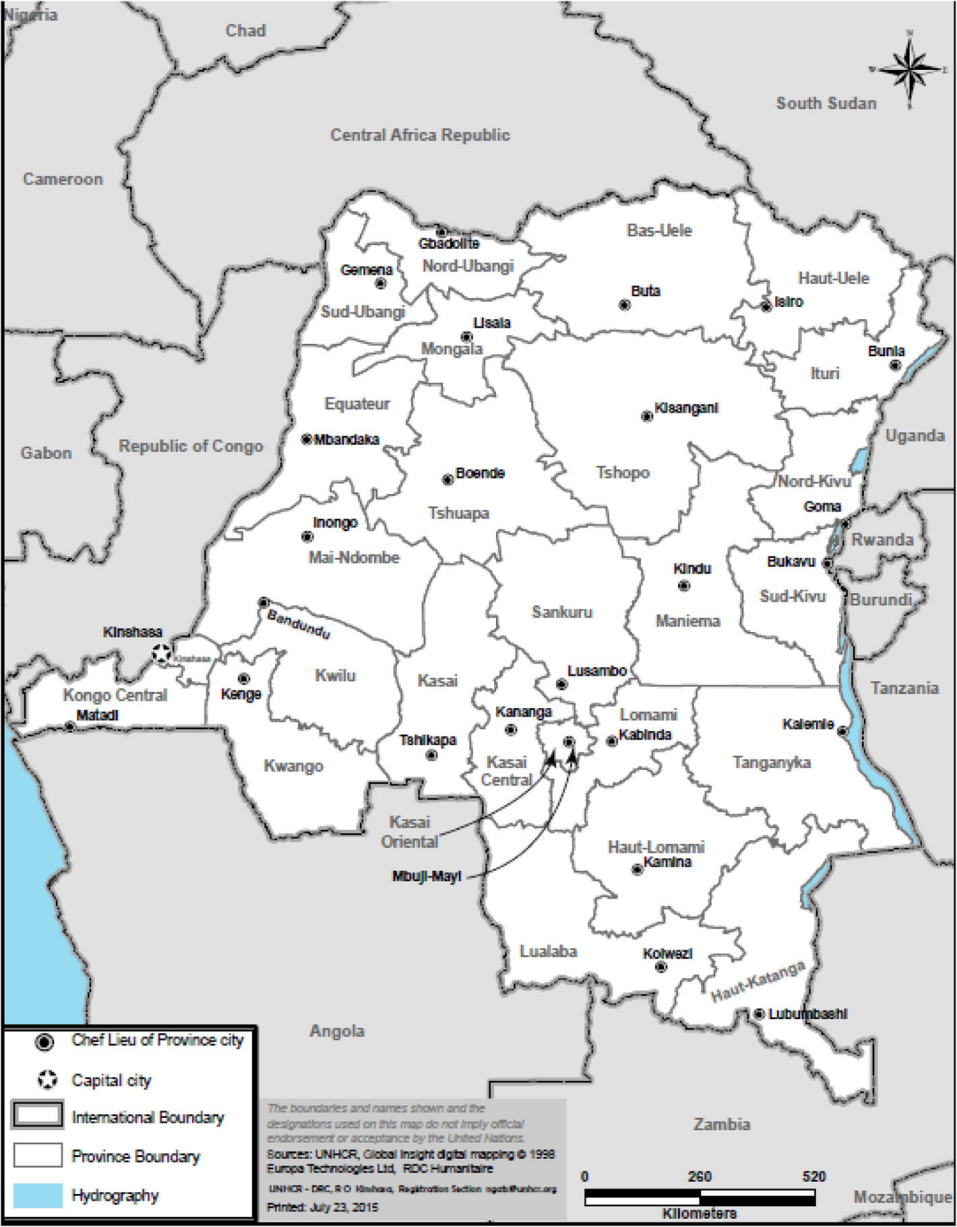
Provinces of the Democratic Republic of Congo

This project was a secondary data analysis of the 2013–2014 Demographics and Health Survey (DHS) for the Democratic Republic of the Congo. The data are representative at the national level for the eleven old and 26 new provinces [20] created in 2015 [11] (Fig. 1).

The DHS, funded by the US Agency for International Development (USAID) with contributions from participating countries, has been administered in over 90 countries across Africa, South & Southeast Asia, Oceania, Latin America & the Caribbean and parts of Eastern Europe [3]. It consists of four general surveys: a household survey, women’s survey, men’s survey, and a biomarker survey [11]. The surveys cover a wide variety of health behaviors and knowledge and are intended to be administered every five years. For purposes of this study tobacco use behaviors were examined, as well as a variety of information on social determinants that were also collected. The dataset supporting the conclusions herein is available in the DHS repository, https://dhsprogram.com/data/available-datasets.cfm.

The survey uses a two-stage cluster sampling process, first with enumeration levels and then selected households; these data are considered to be representative of the population of the country. A complete description of the data collection, cleaning and weighting processes can be found elsewhere [11].

For this analysis we recoded some variable values to reduce the number of small and zero-cell counts. These variables include literacy, occupation and ethnicity. We performed initial single-variable logistic regressions to determine significant relationships between variables. All variables that were significantly related to the dependent measures of interest were then included in final analyses. Logistic regressions predicting smoking, use of snuff and smoking cigars or natural tobacco as dichotomous variables were then conducted. Independent variables included age, educational level, religion, rurality, literacy, wealth index, occupation and ethnicity. Categorical variables were entered as factor variables.

## Results

### Sample

The data for this analysis were drawn from surveys conducted on both men and women. Final samples included 18,827 women and 8656 men. Ages of respondents ranged from 18 to 49 among women (M = 28.3, SD = 9.4) and 18 to 59 for men (M = 31.7, SD 12.3). Frequencies were examined, followed by chi-square analyses and logistic regressions predicting use of three types of tobacco products.

The DHS survey team collected information nominally related to ethnicity. Those ethnic breakdowns however consist, to a large extent, of regional groupings that are not highly reflective of the actual ethnic distribution of people across the country, but rather general geographic regions of the country.

### Prevalence of tobacco use by sex and location

Nationwide, 21.2% of men smoked cigarettes while 0.5% of women smoked cigarettes (Table 1). The prevalence of cigarette smoking among men was highly variable across the country, ranging from 13.5 to 37.7% among males. Cigarette smoking exceeded 35% of the male population in three provinces (Haut-uele, Kasai, Ituri), and exceeded 25% in another three (Equateur, Kasai-central, Bas-uele) (Fig. 1). Cigarette use among females around the country was nearly negligible (under 1%) except for those in Haute-uele (2.5%) and Ituri (4.6%) provinces in the far northeastern corner of the country. Less than 5% of men (4.7%) smoked cigars or natural tobacco wrapped by hand and only 0.4% of women did so. When considering powdered snuff, men’s use levels were highest in Kwango (37.1%) and Kongo central (25.5%) provinces in the very southwestern part of the country. Men’s use levels were also at 17.4% in Kwilu, just east of Kinshasa and 15.1% in Sud Ubangi, in the very northwestern corner of the country. Nationwide, women’t snuff use was only 2.8% but was highest in Tshopo (9.5%), Kwilu (8.8%) and Sud Ubangi (8.9%) and Mai Ndombe (7.2%).

**Table 1:** Percentage use by province

Province	Men Cigarettes	Men Snuff	Men Natural tobacco/cigars	Women Snuff
Kinshasa	13.5	10.1	1.8	1.5
Kwango	19.8	37.1	2.2	10
Kwilu	22.4	17.4	4.2	8.8
Mai-Ndombe	24.9	14.2	7.5	7.2
Kongo Central	23	25.5	3.6	3.7
Equateur	25.7	5.2	5.2	4.7
Mongala	12.2	8.8	5.5	3.9
Nord-ubangi	22.9	1.5	2.6	1.7
Sud-ubangi	22.2	15.1	1	8.9
Tshuapa	26.5	5.9	4.4	4.2
Kasai	36.9	5.3	11.3	2.2
Kasai-central	25.8	0.3	5.2	0.7
Kasai-oriental	21.6	0.3	2.1	0
Lomami	19.8	0.9	7.7	0.6
Sankuru	12.2	2.6	3	0.9
Haut-katanga	20.1	0.4	5.8	0.2
Haut-lomami	16	0.7	4.3	0
Lualaba	21.1	0	15.3	0.2
Tanganyka	20.2	3.1	2.7	0
Maniema	18.1	1	2.4	0
Nord-kivu	17	0.4	6.6	0.1
Bas-uele	31.3	8.2	3	3.9
Haut-uele	35.5	8.2	2.7	2.1
Ituri	37.7	4	15.6	0.7
Tshopo	17.3	9	2.9	9.5
Sud-kivu	13.9	0.6	3.4	0
**Total**	**21.2**	**7.7**	**4.7**	**2.8**

Because the prevalence of tobacco use generally was so low among women – with multiple zero cells in tables – they were excluded from data tables and further analysis. It should be noted, however, that use among Pygmy women is exceedingly high, surpassing 20% using cigarettes and nearly 15% using natural tobacco or cigars. The number of individuals with this appellation, however, was exceedingly small. With this exception, however, tobacco use is generally very low among women in the DRC. Additionally, since prevalence of the use of pipes and chewing tobacco is nearly negligible, those products were also excluded from tables.

### Prevalence of tobacco use by ethnicity

Information on the use of substances in the DRC by varying ethnic groups is difficult to actually ascertain. While the ethnic data collected by the DHS are imprecise due to lack of genomic or haplotype assessments, there is however some advantage in examining these categories when viewed by ethnicity/tribal affiliation which identified some striking difference in tobacco consumption (Table 2). Cigarette smoking was highest among Pygmy men, at 50% while 30% of individuals in the Uele/Lac Albert group smoked. Groups in the southwestern part of the country, including the Bakongo nord & sud (22%) and Bas-kasai & Kwilu (20%) were the highest consumers of snuff.

**Table 2:** Percent of men using tobacco by ethnicity*

Ethnicity	Cigarettes	Snuff	Natural tobacco/cigars
Bakongo Nord & Sud	20	22	2.7
Bas-kasai et Kwilu-Kwango	21.1	20.3	3.2
Cuvette central	21.8	5.7	5.7
Ubangi et Itimburi	20	8.4	2.9
Uele Lac Albert	30	7.7	6.4
Basele-K, Maniema & Kivu	16.5	0.9	4.2
Kasai, Katanga, Tanganyika	21.4	1.3	6
Lunda	28.2	2.6	14.1
Pygmy	56.5	0	21.7
**Total**	**21.1**	**7.7**	**4.7**

*Ethnicity defined broadly by the DHS

#### Social determinants of Tobacco use

We conducted a series of logistic regressions using a variety of social determinants of tobacco use to predict membership in use categories for cigarettes, snuff and natural tobacco/cigars. Results are displayed in Table 3. While use of tobacco was generally predicted by increasing age, living outside of cities, lower education, lower literacy, and poverty demonstrates that different products have different predictive variables.

**Table 3:** Predictors of cigarette smoking among men (*N* = 8583)

	Odds Ratio	se	Z	*p*-value	Lower Limit	Upper Limit
**Age**	1.03	0.003	11.01	0.0000	1.0223	1.0322
**Rurality (Large city ref.)**	1.00	.	.	.	.	.
Small city	0.87	0.163	−0.75	0.4508	0.6011	1.2537
Town	0.71	0.103	−2.38	0.0174	0.5327	0.9412
Countryside	0.73	0.102	−2.26	0.0237*	0.5515	0.9583
**Educational Level (No education ref.)**	1.00	.	.	.	.	.
Primary	1.13	0.161	0.86	0.3893	0.8554	1.4933
Secondary	0.83	0.145	−1.08	0.2797	0.5864	1.1668
Higher	0.43	0.106	−3.42	0.0006	0.2683	0.6993
**Literacy (Cannot read ref.)**	1.00	.	.	.	.	.
Parts of sentence	0.88	0.101	−1.13	0.2568	0.6991	1.1003
Whole sentence	0.70	0.085	−2.91	0.0036	0.5537	0.8907
No card w/ req. lang.	1.47	0.622	0.91	0.3638	0.6407	3.3672
Blind	0.43	0.268	−1.36	0.1746	0.1237	1.4614
**Wealth Index (Poorest ref.)**	1.00	.	.	.	.	.
Poorer	0.82	0.065	−2.54	0.0112	0.6995	0.9551
Middle	0.73	0.060	−3.77	0.0002	0.6245	0.8618
Richer	0.56	0.055	−5.95	0.0000	0.4587	0.6748
Richest	0.31	0.049	−7.37	0.0000	0.2241	0.4202
**Occupation (Agricultural self-employed ref.)**	1.00	.	.	.	.	.
Professional, technical, manager	0.66	0.076	−3.62	0.0003	0.5256	0.8259
Clerical	0.93	0.326	−0.19	0.8456	0.4715	1.8512
Sales	0.79	0.108	−1.70	0.0888	0.6089	1.0356
Not Working	0.24	0.034	−10.05	0.0000	0.1862	0.3223
Agricultural employee	1.07	0.105	0.64	0.5226	0.8777	1.2928
Services	1.37	0.130	3.31	0.0009	1.1372	1.6506
Skilled manual	1.70	0.276	3.26	0.0011	1.2345	2.3343
Unskilled manual	1.54	0.292	2.29	0.0219	1.0649	2.2343
Army	1.57	0.301	2.33	0.0197	1.0741	2.2822
Others	2.00	2.850	0.49	0.6269	0.1223	32.6800
**Ethnicity (Bas-kongo ref.)**	1.00	.	.	.	.	.
Bas-kasai & Kwilu-kwngo	0.91	0.115	− 0.78	0.4348	0.7053	1.1620
Cuvette central	0.79	0.110	−1.68	0.0935	0.6025	1.0402
Ubangi & Itimburi	0.67	0.089	−3.06	0.0022	0.5125	0.8637
Uele Lac Albert	1.08	0.144	0.55	0.5816	0.8281	1.3997
Basele-k, Maniema & Kivu	0.55	0.070	−4.70	0.0000	0.4271	0.7047
Kasai, Katanga, Tanganika	0.86	0.100	−1.31	0.1912	0.6834	1.0790
Lunda	1.10	0.319	0.34	0.7375	0.6249	1.9428
Pygmy	1.69	0.765	1.16	0.2441	0.6980	4.1068
**_cons**	0.44	0.103	−3.51	0.0005	0.2735	0.6928

Variables were tested as significant determinants by conducting individual logistic regressions with each predictor and dependent variable. Those that were not related were not included in further analyses. These included religion, frequency of listening to the radio or frequency of watching television.

Generally, older age predicted smoking, as did living in small towns or the countryside. But age did not have a linear relationship with smoking, with use peaking in the 30–34 age group (30.6%) and then slightly decreasing after. Higher education (post-secondary) was protective. Those who could read an entire sentence had significantly lower odds of smoking. There was a strong linear effect for wealth, with odds of being a smoker in the highest wealth quintile being only 31% of those of the poorest individuals. With regard to occupation, being in a professional, technical or managerial position was highly protective against smoking while being engaged in services, skilled and unskilled manual labor, and the army had significantly greater odds of smoking.

Membership in two groups was significantly protective against cigarette smoking: those in the Ubangi and Itimbiri (far northwest) and the Basele-k, Maniema & Kivu regional groups in the eastern part of the country.

Logistic regression predicting snuff use among males showed only a few significant predictors (Table 4). Age and literacy remained protective while membership in the highest wealth quintile was also protective. With regard to occupation, both unemployment and working in a professional, technical or managerial setting were protective. Those who were unemployed were probably least likely to use snuff because they had no income. All regional ethnic groups had significantly lower snuff use than those in the Bas-kongo area, the reference group.

**Table 4:** Predictors of snuff use among men (*n* = 8583)

	Odds Ratio	Std. Error	z	p-value	Lower Limit	Upper Limit
**Age**	1.02	0.004	5.88	0.0000	1.0149	1.0300
**Rurality (Large city ref.)**	1.00	.	.	.	.	.
Small city	1.02	0.302	0.08	0.9352	0.5750	1.8246
Town	1.00	0.222	−0.02	0.9842	0.6431	1.5413
Countryside	0.96	0.219	−0.19	0.8493	0.6116	1.4991
**Educational Level (No education ref.)**	1.00	.	.	.	.	.
Primary	1.31	0.333	1.05	0.2941	0.7928	2.1535
Secondary	1.60	0.498	1.50	0.1333	0.8668	2.9417
Higher	0.86	0.336	−0.37	0.7077	0.4037	1.8511
**Literacy (Cannot read ref.)**	1.00	.	.	.	.	.
Parts of sentence	0.76	0.152	−1.36	0.1734	0.5142	1.1274
Whole sentence	0.65	0.138	−2.01	0.0442	0.4307	0.9889
No card w/ req. lang.	0.41	0.435	−0.84	0.4010	0.0516	3.2731
Blind	0.47	0.510	−0.70	0.4862	0.0552	3.9656
**Wealth Index (Poorest ref.)**	1.00	.	.	.	.	.
Poorer	0.97	0.125	−0.27	0.7845	0.7496	1.2432
Middle	0.86	0.115	−1.14	0.2525	0.6592	1.1157
Richer	0.77	0.120	−1.66	0.0968	0.5709	1.0474
Richest	0.58	0.145	−2.19	0.0288	0.3536	0.9449
**Occupation (Agricultural self-emp. Ref.)**	1.00	.	.	.	.	.
Professional, technical, manager	0.69	0.113	−2.24	0.0253	0.5036	0.9558
Clerical	0.37	0.280	−1.31	0.1886	0.0863	1.6205
Sales	0.78	0.157	−1.24	0.2138	0.5241	1.1555
Not Working	0.48	0.083	−4.25	0.0000	0.3452	0.6752
Agricultural employee	0.97	0.147	−0.21	0.8327	0.7193	1.3039
Services	0.94	0.150	−0.41	0.6825	0.6841	1.2822
Skilled manual	0.75	0.222	−0.97	0.3339	0.4210	1.3414
Unskilled manual	1.19	0.332	0.63	0.5282	0.6906	2.0576
Army	0.79	0.264	−0.70	0.4828	0.4107	1.5229
Others	1.00	.	.	.	.	.
**Ethnicity (Bas-kongo ref.)**	1.00	.	.	.	.	.
Bas-kasai & Kwilu-kwngo	0.78	0.100	−1.97	0.0491	0.6041	0.9990
Cuvette central	0.16	0.030	−9.68	0.0000	0.1117	0.2337
Ubangi & Itimburi	0.24	0.039	−8.95	0.0000	0.1799	0.3333
Uele Lac Albert	0.21	0.037	−8.88	0.0000	0.1512	0.2994
Basele-k, Maniema & Kivu	0.03	0.008	−12.19	0.0000	0.0148	0.0475
Kasai, Katanga, Tanganika	0.04	0.008	−15.49	0.0000	0.0247	0.0568
Lunda	0.07	0.049	−3.71	0.0002	0.0162	0.2797
Pygmy	1.00	.	.	.	.	.
Other	1.00	.	.	.	.	.
**_cons**	0.24	0.088	−3.87	0.0001	0.1132	0.4892

Hand-rolled tobacco and cigar use was significantly higher in the Basele-k, Maniema & Kivu area as well as the Kasai, Katanga & Tanganyka regions, which are in the eastern and southeastern areas of the country (Table 5). Other than the regional differences, age was again a significant predictor of cigar use, being in the army. Interestingly, being in a town or rural area was protective against using these products. Significant protective factors included secondary or higher levels of education, being in the top three wealth quintiles, and as with other products, working as in professional, technical or managerial positions or sales.

**Table 5:** Predictors of cigar & hand rolled tobacco smoking among men (*N* = 8606)

	Odds Ratio	Std. Error	z	Calculated p-value	Lower Limit	Upper Limit
**Age**	1.03	0.005	5.68	0.0000	1.0169	1.0350
**Rurality (Large city ref.)**	1.00	.	.	.	.	.
Small city	1.09	0.412	0.22	0.8262	0.5169	2.2849
Town	0.53	0.172	−1.96	0.0494	0.2796	0.9984
Countryside	0.77	0.223	−0.89	0.3752	0.4403	1.3624
**Educational Level (No education ref.)**	1.00	.	.	.	.	.
Primary	0.72	0.153	−1.53	0.1250	0.4757	1.0948
Secondary	0.54	0.153	−2.17	0.0300	0.3085	0.9418
Higher	0.13	0.090	−3.01	0.0026	0.0366	0.4967
**Literacy (Cannot read ref.)**	1.00	.	.	.	.	.
Parts of sentence	1.02	0.195	0.12	0.9033	0.7050	1.4855
Whole sentence	0.73	0.153	−1.52	0.1282	0.4792	1.0972
No card w/ req. lang.	2.01	1.071	1.31	0.1916	0.7054	5.7118
Blind	1.82	1.285	0.85	0.3955	0.4568	7.2616
**Wealth Index (Poorest ref.)**	1.00	.	.	.	.	.
Poorer	0.83	0.114	−1.37	0.1707	0.6329	1.0844
Middle	0.65	0.097	−2.89	0.0038	0.4813	0.8688
Richer	0.60	0.113	− 2.70	0.0070	0.4194	0.8714
Richest	0.25	0.089	−3.88	0.0001	0.1241	0.5037
**Occupation (Agricultural self-emp. Ref.**)	1.00	.	.	.	.	.
Professional, technical, manager	0.56	0.141	−2.28	0.0224	0.3458	0.9221
Clerical	0.44	0.453	−0.80	0.4251	0.0592	3.2929
Sales	0.45	0.153	−2.35	0.0189	0.2322	0.8770
Not Working	0.13	0.052	−5.15	0.0000	0.0605	0.2841
Agricultural employee	0.71	0.137	−1.79	0.0736	0.4824	1.0337
Services	1.00	0.177	0.02	0.9828	0.7108	1.4176
Skilled manual	0.65	0.286	−0.97	0.3322	0.2785	1.5405
Unskilled manual	0.58	0.278	−1.13	0.2568	0.2276	1.4845
Army	1.90	0.595	2.05	0.0403	1.0288	3.5096
Others	1.00	.	.	.	.	.
**Ethnicity (Bas-kongo ref.)**	1.00	.	.	.	.	.
Bas-kasai & Kwilu-kwngo	0.98	0.289	−0.05	0.9571	0.5538	1.7495
Cuvette central	1.51	0.442	1.42	0.1544	0.8553	2.6833
Ubangi & Itimburi	0.65	0.200	−1.40	0.1610	0.3555	1.1874
Uele Lac Albert	1.38	0.395	1.11	0.2660	0.7841	2.4143
Basele-k, Maniema & Kivu	1.01	0.281	0.05	0.9619	0.5887	1.7442
Kasai, Katanga, Tanganika	1.73	0.448	2.13	0.0335	1.0439	2.8759
Lunda	3.95	1.661	3.27	0.0011	1.7326	9.0047
Pygmy	2.28	1.366	1.37	0.1692	0.7043	7.3776
Other	1.00	.	.	.	.	.
**_cons**	0.08	0.037	−5.43	0.0000	0.0327	0.2006

## Discussion

The national-level tobacco use data for the DRC are similar to those in many other nations in that tobacco use is highest among those with less education and low literacy [12]. It was also highest among the working poor, with those who were unemployed not consuming tobacco in any form. Since the DHS divides people into income quintiles, the lowest quintile contains both the working poor and unemployed who may have no income at all. Those who had some income appear to have spent it on cigarettes while those with no income could not have, however this question was not explored in the survey. Smoking cigarettes was lowest among those with the highest levels of education and those who worked in professional and managerial positions. Those employed in services, skilled and unskilled manual labor and in the army had significantly higher levels of cigarette smoking. Smoking was generally highest in the cities with use levels dropping as rurality increased. This differs from the findings of [16, 17], who found that tobacco use was higher in rural areas, but it parallels the findings of Pampel [13].

Snuff is another product that is popular in some areas of the country. As with cigarette smoking, use followed predictable patterns of highest use among those with the lowest educational levels and lowest literacy. Again, the unemployed had lower levels of snuff consumption. There were no significant differences in snuff use levels between cities and rural areas. While overall levels of use are small (7% among men), an examination of regional differences showed use rates between zero and 37%. Highest levels of use were found in the southwestern part of the country, as reflected in labeled ethnicities from that area.

Protective factors against using cigars and hand rolled tobacco included living in small towns, increasing education and increased wealth. As with other products, those employed in professional & technical jobs, as well as sales, had significantly less use than the reference group, while being in the army had greater odds of using the products.

### Limitations

This analysis has several limitations. While DHS data are considered to be nationally representative, they may be less so at the sub-national level where the cell sizes of under-represented minorities are too small to elicit confidence in the data. Additionally, “ethnicity” as defined by the DHS, for the most part, is actually a conglomeration of regional ethnic groups aggregated by provinces or regions in selected river basins, and is often a self-described association as compared to associations of groups based on genetic relatedness.

As with any survey, the data are self-reported and may be subject to biases especially among marginalized groups such as the Pygmy tribes. Nevertheless, the data examined in this study are not sensitive and are less prone to socially desirable responses.

## Conclusions

Tobacco use patterns in the DRC generally mirror those seen in other societies. Tobacco remains primarily a product used by the poor and those with the lowest education levels [12, 16, 17]. The nation faces myriad other public health problems, from water and sanitation issues, infectious disease, conflict and internal displacement. Yet tobacco must be recognized as a significant threat to public health and addressed at the ministerial as well as community level to avoid additional needless morbidity and mortality.

As is seen in many other areas of the world, smoking remains a significant concern among the poor and poorly educated, where smoking is generally common among the poorest segments of the population. These groups, already under financial stress, have little disposable income to spend on cigarettes.

Combatting tobacco in Africa remains a difficult task. Tobacco companies are aggressively working to spread their footprint in developing countries [19]. While many countries are signatories to the Framework Convention on Tobacco Control [22], enforcement, in the presence of a variety of other pressing public health problems, remains spotty ([1].) Multi-national tobacco companies also trap growers through predatory loans and the provision of seed and fertilizer that keep them locked in a cycle of producing cheap tobacco [1]. These corporations also work to combat implementation of the Framework Convention on Tobacco Control by bringing trade complaints asserting that attempts to control tobacco are counter to international trade treaties, subjecting countries to various forms of liability and costly legal battles [18].

Given the poverty of many developing countries, more expensive cigarettes that are manufactured in Europe and North America are less often seen, with substitution by locally grown and produced cigarettes. Strong domestic tobacco control that imposes reasonable limits on imports as well as regulates production and sales of domestic product will be essential to improving tobacco control in these countries. All countries are advised to implement the basic steps of MPOWER outlined in the World Health Organization Report on the Global Tobacco Epidemic [25], which incorporate Monitoring tobacco use & prevention policies, Protecting individuals from tobacco smoke, Offering cessation assistance, Warning about the dangers of tobacco use, Enforcing bans on advertising, promotion & sponsorship, and Raising taxes on tobacco. In many LMICs, however, these tactics are both unfeasible and unaffordable for a variety of reasons.

Another area of concern revolves around surveillance and monitoring. Examining only national-level data to ascertain tobacco use levels and patterns may lead to mistaken conclusions that can lead to inefficient and ineffective allocation of resources aimed at controlling tobacco use. National-level datasets provide a picture of the tobacco landscape but because the populations of many countries can be very heterogeneous, tobacco use patterns and burden of disease are not well-represented with such data. For example, consumption of indigenous tobacco and/or cannabis by some tribes is a cultural tradition while for others it is a social adaptation. It has been noted by others [3] that the DRC has about 40 ethnic groups, yet the DHS only recognizes nine plus an “other” group. This means that much of the cultural and ethnic richness is lost in the way that the data are collected. For example, in contrast to the nine general ethnic categories listed in the DHS, the Enquête 1–2-3 sur l’Emploi, le Secteur Informel et les Conditions de Vie des Ménages 2004 [9] specifies 457 unique tribal affiliations with an estimated 213 individual languages in the DRC, 134 that are considered to be “vigorous” [5]. As such, aggregating ethnicities into the nine self-identified groups as done in the DHS poses significant danger for interpretation.

For some variables, in some countries, national-level indicators may be appropriate and helpful in assessing health problems and determining health behaviors. Such information is important in setting national priorities as well as developing appropriate public health interventions. For other variables, though, national-level published data may mask significant disparities in behaviors or problems. Such is the case with the reports regarding tobacco use in the DRC. Tobacco is used disproportionately among the poor, and certain ethnic groups, which highlights that class and culture are likely to interact in a manner to increase the likelihood of tobacco use.

Ministries are well-advised to closely examine tobacco use patterns that consider geographic and ethnic variability as well as other social determinants. Recommendations echo those of [16, 17], who argued that “Tobacco control strategies should target the poor, not/least educated, and agricultural and unskilled workers, who are the most vulnerable social groups in sub-Saharan Africa.”

Additionally, examination of sub-national patterns will lead to more efficient allocation of limited resources and lead to greater improvements in population health. Identification of changing rates of occurrence of diseases known to be related to tobacco can provide indicators that will be important in public health programming. With the continued increase in importance of non-communicable diseases as a significant cause of death in developing countries increasing attention will be needed with regard to appropriate measurement and surveillance.

## Data Availability

The datasets analyzed during the current study are available in the Demographic and Health Surveys repository at https://www.dhsprogram.com/What-We-Do/survey-search.cfm?pgtype=main&SrvyTp=country&ctry_id=243
